# Editorial: Simultaneous multiparametric and multidimensional cardiovascular magnetic resonance imaging

**DOI:** 10.3389/fcvm.2023.1205994

**Published:** 2023-06-05

**Authors:** Aleksandra Radjenovic, Anthony G. Christodoulou

**Affiliations:** ^1^ School of Cardiovascular & Metabolic Health, University of Glasgow, Glasgow, United Kingdom; ^2^ Biomedical Imaging Research Institute, Cedars-Sinai Medical Center, Los Angeles, CA, United States

**Keywords:** magnetic resonance imaging (MRI), heart imaging, cardiac magnetic resonance (CMR), multiparametric imaging, quantitative imaging, SMART CMR, cardiovascular imaging, artificial intelligence-AI

**Editorial on the Research Topic** Simultaneous multiparametric and multidimensional cardiovascular magnetic resonance imaging

Multiparametric quantitative cardiovascular magnetic resonance (CMR) imaging is a powerful tool for evaluating myocardial morphology, function, and tissue status. It provides objective, reproducible measurements appropriate for diagnosing and longitudinally monitoring both focal and diffuse cardiovascular diseases. CMR has unparalleled flexibility: it is sensitive to a wide range of physical and physiological processes such as motion, T1 and T2 relaxation (biomarkers for e.g., fibrosis, edema, and inflammation), blood flow, diffusion, and more. However, the multiparameter sensitivity of CMR is also its weakness, as sequentially targeting each of these individual processes requires an inefficient combination of electrocardiogram (ECG) triggering, respiratory control, and precise pulse sequence timing. This complicated, disjointed imaging paradigm has limited the adoption of multiparametric quantitative CMR to only specialized imaging centers, preventing it from reaching its full potential.

**Figure 1:**
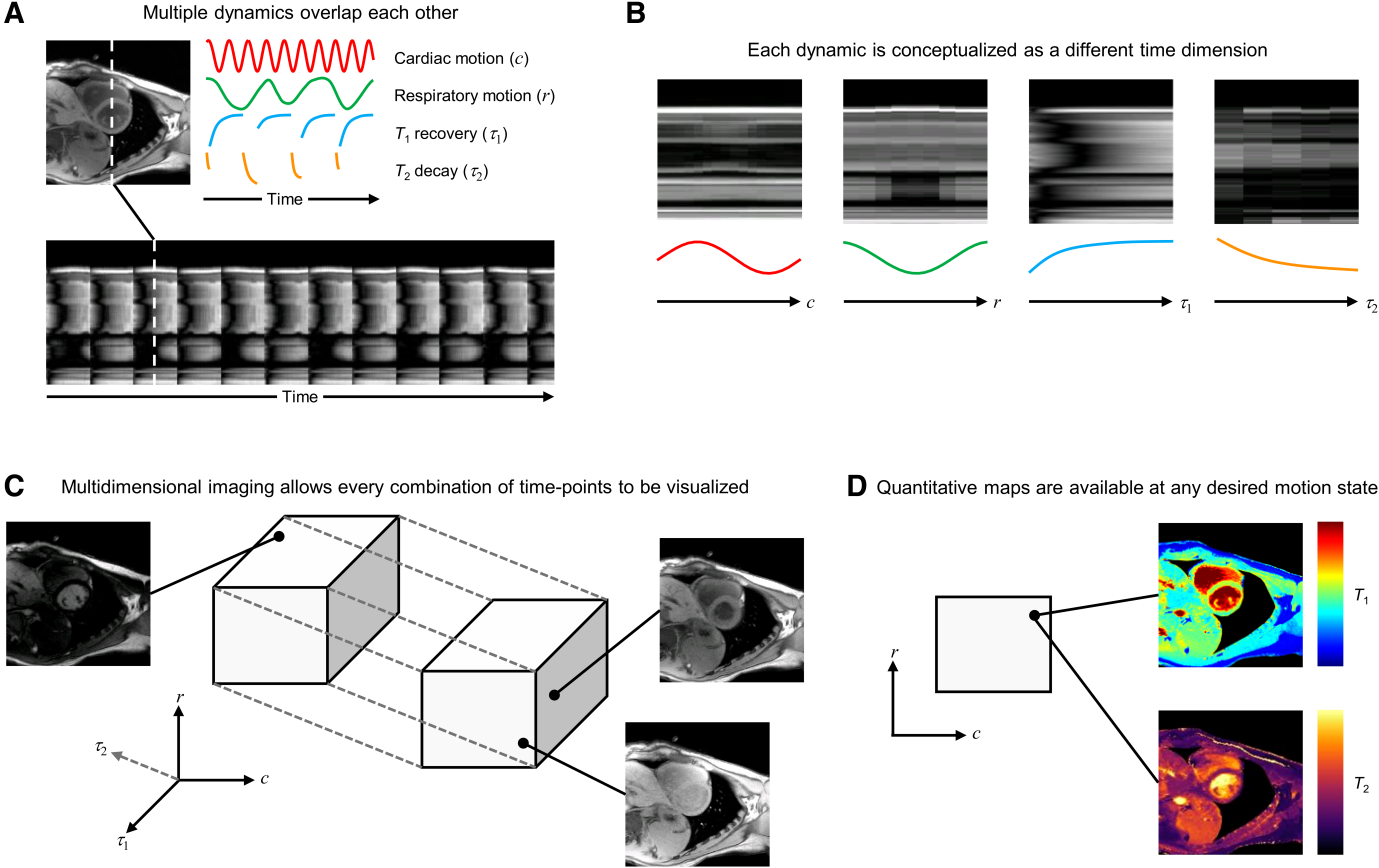
Multidimensional imaging example. (**A**) Continuous data acquisition without electrocardiography (ECG) triggering during free-breathing results in overlapped cardiovascular dynamics. This example shows cardiac motion, respiratory motion, T1 recovery, and T2 decay. (**B**) Multidimensional approaches consider each dynamic as a separate time dimension, disentangling the different dynamics. (**C,D**), The resulting multidimensional dataset enables the visualization of arbitrary combinations of time points (**C**) and can provide co-registered quantitative maps for each cardiac or respiratory motion state (**D**). Figure adapted from Otazo 2018 (2).

In this article collection, we showcase the results of ongoing research in Simultaneous Multiparameter Acquisition and Reconstruction Techniques (SMART) for CMR (1), which will facilitate the transition from the current sequential MR imaging model into a new, single push-button MRI model. This new model is capable of simultaneously capturing multiple types of contrast and quantitative maps of tissue properties from one comprehensive, continuous dataset (Figure 1).

This transition is analogous to moving from sequential “single color” imaging to simultaneous “prismatic” imaging. It has the potential to revolutionize all branches of diagnostic MRI, but its impact will undoubtedly be most profound in the cardiovascular domain. Here, it can eliminate the need for respiratory or ECG triggering or gating, making CMR accessible to patient groups who currently cannot benefit from its full diagnostic potential.

So far, multidimensionality has been used to achieve a better workflow with equivalent results. This in itself can increase access to CMR for patients suffering from various types of arrhythmia, breathing difficulties, and in low- to middle-income countries where technologist training is a burden or scanner throughput is at a premium due to scarce imaging resources. Looking even farther ahead: in addition to improving workflow and access, multidimensional imaging may provide additional value through novel time-resolved quantification and study of interactions between dimensions. Furthermore, the rich multiparametric information in multidimensional CMR images is spatiotemporally co-registered, and therefore ready-made for the development of artificial intelligence tools for diagnosis, risk prediction, therapy monitoring and more.

Another advantage of SMART CMR is its potential to address the issue of reproducibility, another significant barrier to wide adoption for quantitative mapping techniques. Although quantitative maps are ideally absolute biomarkers, differences in cut-off values for healthy and pathological tissues exist across imaging centers. This inconsistency stems from factors like incomplete modeling and variable segmentation, limiting the adoption of these techniques by those not directly involved in their development (Ogier et al.). SMART CMR can potentially improve reproducibility in several ways. For instance, it can provide standardized timing not reliant on patients' ECG signals, use co-registered parameter maps to improve region drawing and segmentation, and employ multiparameter mapping to model confounding factors that affect repeatability, such as B1 + and magnetization transfer. These improvements may lead to more consistent results across imaging centers, ultimately enhancing the clinical utility of these techniques.

This collection of articles contains reports on existing research that is already producing tangible, peer-reviewed scientific results in this emerging field while also offering a projection of what lies in the future, just beyond the horizon.

An overview of simultaneous multiparametric acquisition and reconstruction techniques (SMART) in CMR was provided by Eyre et al. They discuss the theory of SMART CMR, its clinical testing, validation, and examples of how it improves clinical workflows. A further policy and practices review by Fotaki and Velasco et al. (2) highlights the cardiac–liver axis, discussing quantitative MRI methods for non-invasive myocardial and liver tissue characterization in cardiometabolic diseases. It covers current approaches, technical developments, limitations, challenges, and recommendations for clinical validation.

In the realm of cardiac MR fingerprinting (MRF) (3, 4), Hamilton presents deep image prior MRF, a novel reconstruction approach that shortens breathhold and diastolic acquisition window in cardiac MRF, thereby improving scan efficiency and reducing motion artifacts. Liu et al. extend cardiac MRF to simultaneous T1, T2, and proton density fat fraction mapping in the heart through the use of rosette k-space trajectories. Velasco and Fletcher et al. review the latest developments in applying artificial intelligence (AI) to cardiac MRF, discussing how AI optimizes sequences, reduces memory demand, and minimizes computational time for image reconstruction and post-processing.

Expanding the MR Multitasking framework (5), Mao et al. present a simultaneous multi-slice (SMS) technique for motion-resolved, non-ECG, free-breathing T1-T2 mapping, demonstrating its potential for reducing three-slice mapping time without ECG or breath-holds. Building on the TOPAZ technique (6), Weingartner et al. introduce a three-step approach for cardiac phase-resolved LGE imaging, allowing assessment of scar motility and cross-comparison between multiple phases, overcoming limitations of single-phase LGE techniques. Moving to clinical validation, Jarkman et al. evaluate the Multimapping framework (7) for simultaneous myocardial T1 and T2 mapping in patients with a range of cardiovascular diseases, showing high correlation with reference techniques and better image quality in a short breath-hold.

Finally, Axel et al. discuss challenges and opportunities in visualizing and analyzing multidimensional cardiovascular magnetic resonance imaging data, addressing new computational methods, limitations of human perception, and conventional display devices. They suggest that significant breakthroughs in this field may result from exploiting advances in other areas of applied science and technology, such as hyperspectral remote sensing of environment or astronomy.

The articles in this collection present a convincing case that the transition to continuous, multiparametric and multidimensional MRI is within our reach. The question is not if, but when this will happen.
